# Synthesis and Pharmacological Evaluation of Azetidin-2-ones and Thiazolidin-4-ones Encompassing Benzothiazole

**DOI:** 10.4103/0250-474X.45393

**Published:** 2008

**Authors:** B. M. Gurupadayya, M. Gopal, B. Padmashali, Y. N. Manohara

**Affiliations:** Department of Pharmaceutical Chemistry, J. S. S. College of Pharmacy, Mysore-570 015, India; 1 Department of Studies in Biochemistry, Kuvempu University, Shivagangothri, Tholhunase, Davanagere-577 004, India; 2 Sahyadri Science College, Shimoga-577 203, India; 3 Department of Pharmaceutical Chemistry, National College of Pharmacy, Shimoga-577 201, India

**Keywords:** Benzothiazole, azetidinones, thiazolidinones, pharmacological activity

## Abstract

Various 7-chloro-6-fluoro-2-arylidenylaminobenzo(1,3)thiazole (2a-h) have been synthesized by the condensation of 7-chloro-6-fluoro-2-aminobenzo(1,3)thiazole (1) with different aromatic aldehydes. The Schiff's bases on reaction with acetyl chloride, chloroacetyl chloride and phenyl acetyl chloride yielded 1-(7-chloro-6-fluorobenzothiazol-2-yl)-3,4-substituted-aryl-azetidin-2-ones (3a-x). Similarly, cyclization of Schiff's base with thioglycolic acid furnished 3-(7-chloro-6-fluoro-benzothiazol-2-yl)-2-substituted–arylthiazolidin-4-ones (4a-h). The structures of the newly synthesized compounds have been established on the basis of their spectral data and elemental analysis. Some selected compounds were evaluated for antiinflammatory, analgesic, CNS depressant and skeletal muscle relaxant activity.

The β-lactam antibiotics are extensively used for bacterial infections. The cephalosporins^1^ have withstood the onslaught of microorganisms and have come to be physician's arsenal in combating a wide range of microbial infections. Moreover various β-lactams are associated with antitumor^2^, antitubercular^3^, antiinflammatory^4^ activities. Similarly, thiazolidinones have attracted considerable attention as they are also enrolled with wide range of pharmacological activities like anticonvulsant^5^, analgesic^6^ and antiinflammatory^7^ activities. In continuation of our studies on benzothiazole^8^,^9^, we have synthesized benzothiazole moiety linked to bioactive β-lactam and thiazolidinone rings, to analyse their biological profile.

The starting material for the synthesis of desired compounds is 7-chloro-6-fluoro-2-aminobenzo(1,3)thiazole^10^ (1), which on treatment with different aromatic aldehydes in concentrated sulphuric acid yields the respective Schiff bases (2a-h). The Schiff bases were separately reacted with substituted acetyl chloride and mercaptoacetic acid produced 1-(7-chloro-6-fluorobenzothiazol-2-yl)-3,4-substituted-arylazetidin-2-ones (3a-x) and 3-(7-chloro-6-fluoro-benzothiazol-2-yl)-2-substituted-arylthiazolidin-4-ones (4a-h) respectively (Scheme 1). The newly synthesized compounds were characterized by spectroscopic data and elemental analysis and were screened for their antinflammatory, analgesic, CNS depressant and skeletal muscle relaxant activities.

**Scheme 1:**
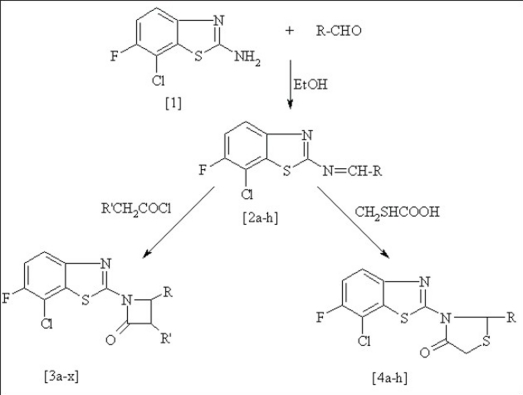
Synthetic scheme of Schiff's bases, azetidine-2-ones and thiazolidin-4-ones R= C_6_H_5_, C_6_H_4_-4-OCH_3_, C_6_H_4_-2-OH, C_6_H_4_-3-OCH_3_, C_6_H_4_-4-N(CH_3_)_2_, C_6_H_4_-2-NO_2_, C_6_H_4_-3Cl and C_4_H_3_O (2-furyl). R' = H, Cl and C_6_H_5_.

## MATERIALS AND METHODS

Melting points were determined in open capillaries and are uncorrected. IR spectra were recorded in KBr on FTIR Shimadzu 1400S and NMR spectra were recorded on AMX-400 in CDCl_3_/DMSO-*d_6_* using TMS as internal standard (chemical shifts in δ ppm). Mass spectra were recorded on FT VG-7070H Mass spectrophotometer using the EI technique at 70 eV. Satisfactory elemental analyses were obtained for all the compounds and were within ±0.4% of the theoretical values. The reactions were monitored on TLC with solvents of varying polarity and the spots were located by iodine vapors. For antiinflammatory and analgesic activities, adult healthy rats of Wistar strain of either sex weighing between 150-200 g were used. For CNS depressant and skeletal muscle relaxant activity studies, adult, healthy mice of Wistar strain of either sex weighing 20-25 g were used. All animals were maintained under standard conditions and had access to pelletted animal feed and water *ad libitum*. The study protocols were carried out as approved by the Institutional Ethics Committee (CPCSEA Reg. No. 144).

To a mixture of 7-chloro-6-fluoro-2-aminobenzo(1,3)thiazole (1) (0.1 mol) and benzaldehyde (0.1 mol), was added two drops of concentrated sulphuric acid and the reaction mixture was refluxed in ethanol (25 ml) for 3 h. The contents were poured into cold water. The Schiff's base (2a) thus formed was filtered off and recrystallised from hot ethanol to give 7-chloro-6-fluoro-(2-hydroxy-benzylidine)-benzo(1,3)thiazole. IR (V_max_): 1650(C=N) and 3480 (Ar-OH). ^1^H NMR(CDCl_3_): 9.25 (s, 1H, -N=CH), 12.1 (s, 1H, Ar-OH), 7.0-8.0 (m, 6H, Ar-H). Mass: m/z 306 and fragment ion peaks at 289, 202, 175 and 140. Similarly, the other Schiff's bases (2b-h) were prepared.

The mixture of Schiff's base (2a) (2.90 g, 0.01 mol) and triethylamine (1.02 ml, 0.01 mol) was dissolved in dioxane (40 ml) and kept in an ice bath. To this, cold solution of acetyl chloride (0.72 ml, 0.01 mol) was added slowly at 0°, stirred for 10-12 h and left over night. The precipitated triethylammonium chloride was filtered off and dioxane was removed by distillation. Residue was poured into cold water; the resulting solid was dried and crystallized from ethanol to give 3a. The Schiff's bases (2b-h) were treated separately with acetyl chloride to get 3b-h. Similarly, 3i-p and 3q-x were prepared by treating 2a-h with chloroacetyl chloride and phenyl acetyl chloride separately. 3i IR (V_max_): 1650(C=O). ^1^H NMR(CDCl_3_):3.7(d, 1H, -NCH), 3.9 (d, 1H, CHCl), 7.2-7.9 (m, 6H, Ar-H); 3q IR(V_max_): 1660 (C=O stretch) azetidinone ring, ^1^H NMR(CDCl_3_): 3.1 (d, 1H, -NCH), 3.7 (d, 1H, CH-Ar), 7.0-7.8 (m, 12H, Ar-H).

A mixture of Schiff's base 2a (2.90 g, 0.01 mol) and mercaptoaceticacid (1.19 ml, 0.01 mol) was dissolved in dioxane (20 ml). A pinch of anhydrous zinc chloride was added and then refluxed for 8 h. Separated solid was filtered, washed with sodium bicarbonate solution and then recrystallised from ethanol. Similarly, the other compounds (4b-h) were prepared. 4a IR (V_max_): 1660 (C=O) thiazolidine ring. ^1^H NMR(CDCl_3_): 3.8 (s, 2H, CH_2_), 3.6 (s, 1H, -NCH), 7.2-7.6 (m, 7H, Ar-H). Mass: m/z 364 and fragment ions peak at 289, 202, 175, 101, 81 and 69. Physical data of the compounds is given in Table 1.

**TABLE 1:** PHYSICAL DATA OF THE SYNTHESIZED COMPOUNDS

Compd No	R	R^1^	Mp (°)	Yield (%)	Mol. formula*
2a	-	-	165	78	C_14_H_8_ClFN_2_S
2b	-	-	160	76	C_15_H_10_ClFN_2_OS
2c	-	-	175	81	C_14_H_7_ClFN_2_OS
2d	-	-	155	69	C_15_H_10_ClFN_2_O_2_S
2e	-	-	180	75	C_16_H_13_ClFN_3_S
2f	-	-	168	72	C_14_H_7_ClFN_3_O_2_S
2g	-	-	170	68	C_14_H_7_C_l2_FN_2_S
2h	-	-	198	70	C_12_H_6_ClFN_2_OS
3a	C_6_H_5_	H	188	50	C_16_H_10_ClFN_2_OS
3b	C_6_H_4_ -4-OCH_3_	H	185	63	C_17_H_12_ClFN_2_O_2_S
3c	C_6_H_4_-2-OH	H	190	58	C_16_H_10_ClFN_2_O_2_S
3d	C_6_H_3_-4-OH, 3-OCH_3_	H	170	63	C_17_H_12_ClFN_2_O_3_S
3e	C_6_H_4_-4-N(CH_3_)_2_	H	195	70	C_18_H_15_ClFN_3_OS
3f	C_6_H_4_-2-NO_2_	H	179	80	C_16_H_9_ClFN_3_O_3_S
3g	C_6_H_4_-3-Cl	H	180	73	C_16_H_9_C_l2_FN_2_OS
3h	C_4_H_3_O (2-furyl)	H	205	68	C_15_H_8_ClFN_2_O_2_S
3i	C_6_H_5_	Cl	150	72	C_15_H_9_ClFN_2_OS
3j	C_6_H_4_-4-OCH_3_	Cl	200	69	C_15_H_11_C_l2_FN_2_OS
3k	C_6_H_4_-2-OH	Cl	194	55	C_16_H_9_Cl_2_FN_2_O_2_S
3l	C_6_H_3_-4-OH, 3-OCH_3_	Cl	187	62	C_17_H_11_C_l2_FN_2_O_3_S
3m	C_6_H_4_-4-N(CH_3_)_2_	Cl	198	65	C_18_H_14_C_l2_FN_3_OS
3n	C_6_H_4_-2-NO_2_	Cl	202	71	C_16_H_8_C_l2_FN_3_OS
3o	C_6_H_4_-3-Cl	Cl	170	82	C_16_H_8_Cl_3_FN_2_OS
3p	C_4_H_3_O (2-furyl)	Cl	210	72	C_14_H_7_C_l2_FN_2_O_2_S
3q	C_6_H_5_	C_6_H_5_	215	82	C_22_H_14_ClFN_2_O_2_S
3r	C_6_H_4_-4-OCH_3_	C_6_H_5_	216	73	C_23_H_16_C_l2_FN_2_O_2_S
3s	C_6_H_4_-2-OH	C_6_H_5_	210	54	C_22_H_16_ClFN_2_O_3_S
3t	C_6_H_3_-4-OH, 3-OCH_3_	C_6_H_5_	235	63	C_22_H_16_ClFN_2_O_3_ S
3u	C_6_H_4_-4-N(CH_3_)_2_	C_6_H_5_	208	68	C_24_H_19_ClFN_3_O_3_S
3v	C_6_H_4_-2-NO_2_	C_6_H_5_	193	75	C_22_H_13_ClFN_3_O_3_S
3w	C_6_H_4_-3-Cl	C_6_H_5_	218	70	C_22_H_13_C_l2_FN_2_OS
3x	C_4_H_3_O (2-furyl)	C_6_H_5_	219	73	C_20_H_12_ClFN_2_O_2_S
4a	C_6_H_5_	-	191	82	C_16_H_10_ClFN_2_OS_2_
4b	C_6_H_4_-4-OCH_3_	-	118	85	C_17_H_12_ClFN_2_O_2_S_2_
4c	C_6_H_4_-2-OH	-	180	80	C_16_H_9_ClFN_2_O_2_S_2_
4d	C_6_H_3_-4-OH, 3-OCH_3_	-	157	84	C_17_H_12_ClFN_2_O_3_S_2_
4e	C_6_H_4_-4-N(CH_3_)_2_	-	142	78	C_18_H_15_ClFN_3_OS_2_
4f	C_6_H_4_-2-NO_2_	-	175	74	C_16_H_9_ClFN_3_OS_2_
4g	C_6_H_4_-3-Cl	-	170	70	C_16_H_9_C_l2_FN_2_OS_2_
4h	C_4_H_3_O (2-furyl)	-	186	65	C_14_H_8_ClFN_2_O_2_S_2_

* C, H, N and S are within the limit of ± 0.3%

### Antiinflammatory activity:

Antiinflammatory activity of some selected compounds was evaluated using carrageenan induced rat hind paw oedema method^11^. The animals were divided into control, standard and test groups, each consisting of six animals. The first group was treated with Tween-80 (1%) suspension which served as control, second group was administered with a dose of 20 mg/kg suspension of diclofenac sodium intraperitoneally which served as standard and other groups were treated with 30 mg/kg of suspension of test compounds in Tween-80. After 30 min, the rats were injected with 0.1 ml of carrageenan (1% w/v) to the sub plantar region of left paw of the rats. The volume of paw was measured using potassium permanganate solution displacement technique with the help of plethysmograph both in control and animals treated with standard and test compounds at 0, 1, 2 and 3 h after injection of carrageenan. The percentage inhibition of oedema was calculated by using formula, percent inhibition = (1–Vt/Vc)×100, where Vt is the mean paw volume of the test drug, Vc is the mean paw volume of the control. The results are recorded in Table 2.

**TABLE 2:** RESULTS OF ANTIINFLAMMATORY ACTIVITY OF THE SYNTHESIZED COMPOUNDS

Compound No.			Paw volumes ±SEM and% reduction			
	
	1 h		2 h		3 h	
			
	Mean±SEM	%RPEV	Mean±SEM	%RPEV	Mean±SEM	%RPEV
3a	1.50±0.07	14.60	1.74±0.07	17.14	1.87±0.06*	22.08
3c	1.43±0.03	19.66	1.53±0.05	27.14	1.70±0.04*	29.16
3f	1.40±0.05	21.34	1.45±0.05*	30.95	1.63±0.04*	32.08
3i	1.42±0.03	20.22	1.57±0.11	25.23	1.63±0.10*	32.08
3k	1.38±0.05	22.47	1.40±0.06	33.33	1.43±0.05*	40.41
3n	1.32±0.07	25.84	1.35±0.05	35.71	1.35±0.04*	43.75
3q	1.50±0.04	15.73	1.78±0.05	15.23	1.90±0.02	20.83
3s	1.38±0.05	22.47	1.70±0.05	14.04	1.80±0.05*	25.00
3v	1.30±0.03	26.96	1.65±0.03	21.42	1.75±0.06*	27.08
4a	1.48±0.04	22.00	1.57±0.05*	25.23	1.65±0.06*	31.25
4c	1.45±0.04	18.50	1.53±0.04	27.14	1.63±0.06	32.08
4f	1.28±0.03	28.08	1.42±0.06*	32.38	1.48±0.04*	38.30
Control	1.78±0.05	-	2.10±0.05	-	2.4±0.03	-
Diclofenac Sodium	0.99±0.05	43.24	1.11±0.03*	46.00	1.11±0.05*	54.00

RPEV is reduction in paw edema volume

* Indicates significant difference at p<0.001 when compared to control

### Analgesic activity:

The animals were divided into four groups of six animals each. The animals, which showed reaction time of 2-3 s, were selected for experiment and analgesic activity of some selected compounds was studied by tail flick method^12^. The tail received radiant heat from a wire, which is heated by passing a current of 6 mA. The time taken for the withdrawal of tail was recorded before the administration of the compounds and for 30, 60, 90 and 120 min after administration of compounds. The cut off time for determination of latent period was taken as 40 s to avoid the injury to the skin and based on our pilot studies. One group served as a standard (pentazocine hydrochloride) with dose of 10 mg/kg body weight and another group served as control (1% Tween-80) and rest of the groups used for the test drugs. The test compounds and pentazocine hydrochloride were suspended in 1% Tween-80 which was used as vehicle for the control group. The tested compounds were administered at the dose of 30 mg/kg in the form of suspension and administered intraperitoneally. The results of analgesic activity are shown in Table 3.

**TABLE 3:** RESULTS OF ANALGESIC ACTIVITY OF THE SYNTHESIZED COMPOUNDS

Compound No.	ABRT in sec	ABRT in sec after treatment			
		
	Pre treatment ‘0’ min	15 min	30 min	45 min	60 min
3a	3.60±0.08	4.5±0.06*	6.53±0.04*	7.03±0.13*	11.20 ±0.2*
3c	3.80±0.02	4.5±0.06*	7.05±0.08*	11.40±0.2*	11.40 ±0.2*
3f	3.60±0.02	4.62±0.10*	4.67±0.06*	5.50±0.14*	5.30±0.03*
3i	3.20±0.04	5.5±0.22*	5.5±0.22	4.4±0.20	3.40±0.37
3k	3.40±0.10	3.83±0.31	6.83±0.48*	6.00±0.37*	6.50±0.22*
3n	2.62±0.04	2.67±0.21	3.33±0.21	4.67±0.21	4.00± 0.37
3q	3.60±0.10	4.17±0.31*	5.17±0.31	8.0±0.26	9.83±0.31*
3s	3.20±0.15	4.00±0.26*	5.5±0.34	6.17±0.31*	6.67±0.21*
3v	3.50±0.02	5.33±0.33*	8.83±0.78*	5.67±0.21*	5.33±0.21*
4a	3.10±0.06	7.17±0.48*	11.17±0.40*	10.67±0.5*	9.67±0.21*
4c	3.60±0.06	6.33±0.80*	7.76±0.04	10.33±0.12*	9.25±0.08
4f	3.60±0.20	3.67±0.21*	3.83±0.31	4.33±0.22	4.33±0.42
Control	3.60±0.06	3.60±0.10*	3.50±0.15	3.60±0.15	3.40±0.06
PentazocineHCl	3.60±0.08	4.93±0.18*	8.90±0.15*	10.10±0.2*	12.50±0.1*

ABRT: Average Basal Reaction Time

* Indicates significant difference at p<0.001 when compared to control

### CNS depressant activity:

The CNS depressant activity^13^ of the compounds was studied on mice using actophotometer. Animals of either sex weighing 25-30 g were divided into groups of six each. The actophotometer counts for 10 min were recorded by placing the animals in the actophotometer, which gives the initial reading. The compounds were administered intraperitonially in the form of suspension prepared in 1% Tween-80 at a dose of 30 mg/kg body weight. Each group served as its own control. Diazepam was administered as standard to one of the test group at a dose of 5 mg/kg body weight. After 30 min and 60 min of administration of test compound, the actophotometer counts were noted for 10 min. Decrease in the number of counts for each group was recorded and finally, the percentage CNS depressant activity was determined. The results of CNS depressant activity are shown in Table 4.

**TABLE 4:** RESULTS OF CNS DEPRESSANT ACTIVITY OF THE SYNTHESIZED COMPOUNDS

Compound	Before drug (Mean±SEM)	After drug (Mean±SEM)		CNS depressant activity (%)	
			
		30 min	60 min	30 min	60 min
3a	835.91±03.63	438.00±07.53	621.50±09.45	46.50	23.60
3c	830.90±03.63	113.17±07.00*	354.33±04.12*	86.20	56.40
3f	804.65±02.60	272.33±14.55*	503.83±04.94	67.00	38.00
3i	877.65±02.50	324.17±05.30*	386.83±06.43*	60.40	52.50
3k	915.63±02.30	384.17±14.28*	453.33±13.24*	53.10	44.30
3n	858.13±05.36	360.50±18.36*	466.17±12.23*	56.00	42.70
3q	766.18±07.21	477.17±07.82*	655.83±12.08	41.70	19.30
3s	811.55±02.20	252.50±16.80*	347.00±17.80*	69.20	57.30
3v	823.37±01.45	608.33±02.23	725.33±02.94	25.70	10.80
4a	811.50±02.00	624.00±07.13	733.50±03.45	23.80	09.80
4c	825.53±02.14	215.00±15.92*	405.00±08.37*	73.70	50.20
4f	815.49±01.93	217.33±08.73*	385.33±12.12*	73.50 52.60
Control	820.23±06.88	818.67±05.93	813.17±07.01	-	-
Diazepam	840.64±09.26	82.17±04.04*	112.50±06.55*	90.00	86.20

* Indicates significant difference at p<0.001 when compared to control

### Muscle relaxant activity:

Some of the synthesized compounds were also tested for muscle relaxant activity^14^ (muscle grip strength) in mice using rotorod apparatus. The animals were trained to remain on the rotating rod (moving at speed of 25 rpm) for 5 min. The animals which were able to remain on the rotating rod for 5 min and above were selected for study and were divided into groups of six each. The compounds were administered intraperitonially at a dose of 30 mg/kg body weight in the form of suspension in 1% Tween-80 while one group received diazepam as a standard drug at a dose of 4 mg/kg body weight. After 30 min, the mice were placed again on the rotating rod and animal's fall off time was recorded. The percentage muscle relaxant activity was calculated and the results are shown in Table 5.

**TABLE 5:** RESULTS OF SKELETAL MUSCLE RELAXANT ACTIVITY OF SYNTHESIZED COMPOUNDS

Compound	Fall of time		% Decrease in time
		
	Before the drug treatment	After the drug treatment	
3a	43.00±1.37	15.50±0.12*	64.00
3c	23.50±1.52	14.17±1.01*	39.70
3f	26.17±1.19	16.00±0.68*	38.86
3i	38.50±1.38	22.50±1.38*	41.55
3k	26.83±1.47	20.67±0.95	22.95
3n	34.83±1.35	16.67±0.80*	52.13
3q	44.00±1.65	33.67±0.33	23.50
3s	35.67±1.25	30.62±1.01	14.15
3v	42.33±0.76	38.17±1.28	09.30
4a	32.67±0.84	15.33±1.05*	53.07
4c	26.17±1.08	10.83±1.38*	58.61
4f	30.17±0.95	12.00±1.10*	60.22
Control	34.83±1.25	34.67±1.33	-
Diazepam	28.32 ± 0.20	5.75 ± 0.07*	79.69*

* Indicates significant difference at p<0.001 when compared to control

## RESULTS AND DISCUSSION

Two new series of compounds namely substituted azetidinones (3a-x) and thiazolidinones (4a-h) possessing fluoro-benzothiazoles have been synthesized by using experimental protocol as shown in Scheme 1. All the derivatives were supported by spectral data. The IR, ^1^H-NMR and Mass spectra are in agreement with the proposed structures.

The result of the antiinflammatory activity summarized in Table 2 indicated that 3-chloro-azetidinones showed better activity among the tested compounds. Further the presence of ortho substituted aryl ring at 4^th^ position of azetidinones moiety increased the anti inflammatory activity. Substitution of chloro group with phenyl ring at 3^rd^ position of the azetidinone generally causes decrease in the activity. Among the thiazolidinones, 2-nitro phenyl attached to 5^th^ position of thiazolidinone ring showed maximum activity.

The analgesic activity summarized in Table 3, indicated that 3a and 3c showed maximum analgesic activity. The compounds 4a and 4c exhibited higher analgesic activity than the standard pentazocine at 15, 30 and 45 min interval. However the activity decreases after 60 min suggesting shorter duration of activity for the same.

The CNS depressant activity (Table 4) reveals that 2-hydroxyl phenyl substituted azetidinone (3c) and thiazolidinone (4c) showed better depressant activity. However activity decreases after 60 min indicating that the molecules to be short acting CNS depressants. The skeletal muscle relaxant activity (Table 5) indicated that the thiazolidinones (4a, 4c and 4f) and azetidinone (3a) showed significant muscle relaxant activity.
